# Vitamin D Supplementation Improves Mitochondrial Function and Reduces Inflammation in Placentae of Obese Women

**DOI:** 10.3389/fendo.2022.893848

**Published:** 2022-05-31

**Authors:** Elysse A. Phillips, Nora Hendricks, Matthew Bucher, Alina Maloyan

**Affiliations:** ^1^ Knight Cardiovascular Institute, Oregon Health & Science University, Portland, OR, United States; ^2^ Department of OB/GYN, Oregon Health and Science University, Portland, OR, United States

**Keywords:** maternal obesity, Vitamin D, cord blood, maternal blood, placenta, mitochondrial function, placental inflammation

## Abstract

**Background:**

About 30% of women entering pregnancy in the US are obese. We have previously reported mitochondrial dysregulation and increased inflammation in the placentae of obese women. Vitamin D (VitD) is a major player in calcium uptake and was shown to modulate mitochondrial respiration and the immune/inflammation system. Studies show decreased VitD levels in obese individuals; however, the effect of maternal obesity on VitD metabolism and its association with placental function remains understudied.

**Methods:**

Maternal and cord blood plasma and placental samples were collected upon C-section from normal-weight (NW, body mass index [BMI]<25) and obese (OB, BMI>30) women with uncomplicated pregnancies at term. We measured 25(OH)D_3_ (calcidiol) levels in maternal and cord blood plasma using ELISA. We assessed the expression of CYP27B1, an activator of calcidiol, and Vitamin D receptor (VDR) in placentae from NW and OB, and women with gestational diabetes and preeclampsia. In addition, we examined the effects of VitD supplementation on mitochondrial function and inflammation in trophoblasts from NW and OB, using the Seahorse Bioanalyzer and Western blot, respectively.

**Results:**

Vitamin D levels in blood from OB but not NW women and in cord blood from babies born to NW and OB women showed a significant inverse correlation with maternal pre-pregnancy BMI (r=-0.50, *p*<0.1 and r=-0.55, *p*=0.004 respectively). Cord plasma VitD levels showed a positive correlation with placental efficiency, i.e., the ratio between fetal and placental weight, as well as with maternal blood VitD levels (r=0.69 and 0.83 respectively, *p*<0.00). While we found no changes in CYP27B1 in OB vs. NW women, VDR expression were decreased by 50% (*p*<0.03) independent of fetal sex. No changes in VDR expression relative to BMI-matched controls were observed in the placentae of women with gestational diabetes or preeclampsia. Cytotrophoblasts isolated from placentae of OB women showed a dose-dependent increase in VDR expression after 24-hour treatment with calcitriol (10 nM and 100 nM), an active form of VitD. Trophoblasts isolated from OB women and treated with calcitriol improved mitochondrial respiration (*p*<0.05). We also found a two-fold increase in expression of the NLRP3 inflammasome and the pro-inflammatory cytokine IL-18 in trophoblasts isolated from placentae of OB women (*p*<0.05), with IL-18 expression being reversed by calcitriol treatment (100 nM).

**Conclusions:**

We show that VitD deficiency is at least partially responsible for mitochondrial dysfunction and increased inflammation in the placentae of obese women. Vitamin D supplementation could be beneficial in improving placental dysfunction seen in obese women.

## Introduction

Epidemiological studies have shown that obese individuals have lower levels of circulating Vitamin D (VitD) compared with normal-weight people (1), though the mechanism for this has not yet been extensively studied. Vitamin D is a lipid-soluble vitamin that performs many important functions in the human body, including aiding in calcium absorption (2), exerting antimicrobial properties (3), and helping to reduce inflammation (4). Once VitD is obtained, either through sunlight or the diet, it enters the bloodstream, where it is transported to the liver for hydroxylation to its inactive form, calcidiol [25(OH)D_3_], and subsequently to the kidney for its final hydroxylation by cytochrome P450 family 27 subfamily B member 1 (CYP27B1) to the active form, calcitriol [1a,25(OH)_2_D_3_] (5). During pregnancy, VitD plays important roles in supporting calcium absorption and the healthy growth of bones in the developing fetus; it also aids immune system function (6).

In the United States, it is estimated that over one-third of the adult population is obese, defined as having a body mass index (BMI) greater than 30 (7). More than 65% of women entering pregnancy in the US are either overweight or obese. Pregnancies in obese mothers generate an adverse intrauterine environment *via* both their inflammatory milieu (8) and metabolic and endocrine derangements (9). Obesity impacts the outcome of the pregnancy *per se*, and is associated with hypertensive disorders, gestational diabetes, preeclampsia, and thromboembolic events (10). It also affects the fetus and newborn, causing congenital malformations, large- and small-for-gestational-age infants, and stillbirth. Importantly, maternal obesity leads to cardiovascular dysregulations (11–13) and to obesity and metabolic diseases in offspring (10). In addition, data also show that maternal obesity results in metabolic inflammation in the mother and programs inflammation in the offspring (14).

The placenta is now recognized as a critical regulator of fetal growth and development and as the transducer for communication of maternal and uterine environments to the fetus, whether normal or adverse (15). Thus, despite its short lifespan, the placenta plays a crucial role in offspring health. Several groups including our own have shown that maternal obesity compromises placental function, even in pregnancies having apparently “normal” outcomes (10, 16–18). In particular, maternal obesity has been reported to dysregulate placental metabolism, leading to abnormal mitochondrial respiration and excessive production of reactive oxygen species (16), accumulation of inflammation (19), dysregulated autophagy (17), and changes in miRNA expression (20) and epigenetic modifications (21).

At the molecular level, placentae from obese women were demonstrated to have increased expression of the nucleotide-binding oligomerization domain, leucine-rich repeat-, and pyrin domain-containing 3 (NLRP3) inflammasome, a key mediator of sterile inflammation (22). Inflammasomes are multiprotein complexes, assembly of which is triggered by both microbial and endogenous danger signals and results in activation and cleavage of caspase-1. When activated, caspase-1 cleaves gasdermin D (23) to generate an N-terminal cleavage product that triggers inflammatory cell death (pyroptosis) and the release of pro-inflammatory cytokines interleukin-1β (IL-1β) and IL-18 (24). Excessive activation of the NLRP3 inflammasome has been shown to lead to diabetes, atherosclerosis, and obesity-induced insulin resistance (25). Vitamin D has been found to inhibit the NLRP3 inflammasome either by direct binding between NLRP3 and the Vitamin D receptor (VDR) (26) or *via* VDR signaling-mediated inhibition of cytokine secretion (27). However, the specific role of VitD in maternal obesity-mediated placental inflammation remains understudied.

The aim of this study was to determine whether obesity in pregnancy affects circulating Vitamin D levels. Using maternal and umbilical cord blood collected from normal-weight and obese women at term, we found maternal BMI-dependent changes in the levels of VitD and its receptor. We also assessed the role of VitD supplementation in addressing the placental mitochondrial dysfunction and increased placental inflammation seen in the setting of maternal obesity.

## Materials and Methods

### Ethics Statement

The studies involving human participants were reviewed and approved by the Institutional Review Board of Oregon Health & Science University. Maternal blood, cord blood, and placental tissue were collected from labor and delivery units with informed consent from the patients.

### Collection and Processing of Placental Tissue

Placentae were collected at term, immediately after elective C-section (gestational weeks 39-40) with no labor. C-sections deliveries prior to labor were chosen because uterine contractions during labor have been shown to be associated with activation of oxidative stress and pro-inflammatory signaling, with increased accumulation of COX-2, TNFα, and IL-1β as well as stabilization of HIF-1α in placental tissue (28). In addition, the duration of labor increases the severity of the impact, which is to be expected. This study was supported by report from Lager et al. (29). These findings indicate that in many respects a vaginally delivered placenta does not accurately reflect the organ’s normal *in vivo* state and caution against the use of such placentae for biochemical and molecular studies. Placental tissues for this study were collected from subjects who: 1) Had a singleton pregnancy; 2) Delivered by C-section; 3) Had either normal or high pre-pregnancy body mass index (BMI), respectively grouped as normal-weight (NW; BMI=18.5-24.9) or obese (OB; BMI=30-45); 4) Had either uncomplicated pregnancy or developed gestational diabetes A2GDM controlled by insulin; and 5) Were either normotensive or developed severe preeclampsia defined as presence of hypertension (systolic blood pressure >160 mmHg and/or diastolic blood pressure >110 mmHg on two occasions 2 - 240 hours apart) and proteinuria (≥2 protein on dipstick) occurring after 20 weeks of gestation in a previously normotensive woman. Exclusion criteria included multifetal gestation; pre-gestational chronic inflammatory diseases (asthma, type 2 diabetes, rheumatoid arthritis, Crohn’s disease, etc); use of tobacco or illicit drugs, or both; and recent bariatric surgery. The placentae were randomly sampled as described previously (30). Placental efficiency was calculated as the ratio between birth weight and placental weight.

### Plasma Collection

Maternal blood was collected from fasting patients before C-section. Cord blood was collected and placed in EDTA-containing collection tubes. Plasma was immediately separated from whole blood by centrifugation at 2000 g for 10 min at 4°C, then flash-frozen in liquid nitrogen and stored at -80°C for further analyses.

### Materials

ELISA kits to detect circulating 25(OH) Vitamin D (calcidiol) were purchased from Abcam (Cambridge, MA). Antibodies against Vitamin D receptor (VDR) and against mouse and rabbit IgG were purchased from Cell Signaling Technology (Danvers, MA), and the antibody against cytochrome P450, family 27, subfamily B, polypeptide 1 (CYP27B1) was purchased from ThermoFisher (Waltham, MA). Anti-NLRP3 was purchased from Novus Biologicals (Centennial, CO), and antibodies against gasdermin D and anti-IL-18 were purchased from Biolegend (San Diego, CA). Antibodies against caspase-1 that recognized both total and cleaved forms were purchased from Cell Signaling, and the antibody against endogenous control β-actin (ACTB) was purchased from Sigma-Aldrich St. Louis, MO). Pierce™ BCA Protein Assay Kits were also purchased from ThermoFisher. For cell culture experiments, calcitriol was purchased from Cayman Chemical Company (Ann Arbor, Michigan). Mitochondrial inhibitors oligomycin, FCCP, antimycin A, and rotenone were purchased from Sigma.

### Maternal Serum and Cord Blood Vitamin D Content

Circulating levels of 25(OH) Vitamin D were measured using an enzyme-linked immunosorbent assay (ELISA) according to manufacturer instructions.

### Tissue Processing and Sampling

Villous tissue was dissected from placentae and flash-frozen in liquid nitrogen for protein expression measurements. Cytotrophoblasts (CTBs) were isolated by enzymatic digestion followed by density gradient purification as we previously described (30). CTBs were plated in Iscove’s Modified Dulbecco’s Medium (ThermoFisher Scientific, catalog # 12440), containing 25 mM D-glucose (dextrose), and supplemented with 10% FBS and penicillin/streptomycin. Cells were allowed to fuse and form a multinucleated syncytium for 72 hours before measurements were taken.

### Protein Isolation

Proteins were isolated from flash-frozen villous tissue or from cytotrophoblasts that were cultured for 72 hours. Samples were lysed using ice-cold radioimmunoprecipitation assay (RIPA) buffer with freshly-added protease and phosphatase inhibitors. The resulting mixture was transferred to a 1.5 ml tube and centrifuged at 1000 x *g* for 10 minutes at 4°C to remove cellular debris. The supernatant was transferred to a fresh tube and protein concentration was quantified using a Pierce Bicinchoninic Acid (BCA) Protein Assay Kit.

### Western Blotting

About 25 μg of protein was separated on a 4-20% SDS-PAGE gel, then transferred to a polyvinylidene difluoride (PVDF) membrane and blocked for one hour in 5% (w/v) milk in TBS solution with 0.1% Tween 20. Membranes were subsequently incubated with primary antibodies, then washed, blocked in 1% milk, and probed with secondary antibodies conjugated to HRP. Western blot membranes were visualized using the G: Box from Syngene (Frederick, MD) and analyzed using Genetools software (Syngene). All samples were normalized to β-actin (ACTB).

### Cell Culture Experiments

Cytotrophoblasts (CTB) were isolated from term placentae collected at C-section. At 24 hours after plating, the CTBs were treated with two concentrations (10 nM and 100 nM) of the active form of Vitamin D (calcitriol) for 24 hours. CTBs were then cultured another 24 hours for a total of 72 hours, after which the cells were harvested for protein detection by Western blot. Mitochondrial respiration in isolated trophoblasts was assessed using a Seahorse Bioscience XFe96 analyzer (Agilent, USA).

### Mitochondrial Respiration Assay

Twenty-four hours before mitochondrial respiration was measured, a sensor cartridge was calibrated using Seahorse Calibrant Solution (Agilent, CA) and placed in a non-CO_2_ incubator overnight. One hour before the assay, complete Iscove’s Modified Dulbecco’s Medium (IMDM) media was exchanged for Seahorse base media supplemented with 25 mM glucose, 4 mM glutamine, and 1 mM pyruvate (same as culture media). Cells were then placed in a non-CO_2_ incubator for one hour and were allowed to equilibrate before the oxygen consumption rate (OCR) was measured using a Seahorse XFe96 Analyzer. Basal respiration was calculated from four baseline OCR readings. ATP-coupled respiration, maximum respiration, spare capacity, proton leak, and non-mitochondrial respiration was calculated from OCR readings following the injection of oligomycin (1 μM), FCCP (1 μM), and a mixture of rotenone (3 μM) and antimycin A (1.5 μM) as we described previously (16). The effect of Vitamin D treatment was calculated by taking the ratios of parameters in treated and untreated cells. All assays were normalized to total DNA content per well using the Quant-iT Picogreen dsDNA assay kit.

### Statistical Analysis

The Shapiro–Wilk test was used to test for normal distribution in all data sets. For normally distributed data, significant differences between groups were tested as an interaction between fetal gender (M, F) and pregnancy complications such as maternal obesity with a two-way ANOVA followed by Student’s *t*-test. For non-parametric data, the Kruskal–Wallis test was applied for the same factors (fetal sex and maternal adiposity), followed by the Mann–Whitney U *post hoc* test. Data from males and females were pooled when no fetal sex-dependent differences were observed. *P*-values < 0.1 are reported as statistically significant.

## Results

### Clinical Characteristics of Study Participants

**Table 1** shows the clinical characteristics of the patients participating in this study. By experimental design, maternal BMI was significantly different between the groups. The gestational weight gain in OB mothers with female fetuses was significantly smaller than that in NW mothers (*p*<0.05). No significant differences in maternal age, gestational age, birth weight, or placental weight were detected between the groups.

**Table 1:** Clinical characteristics of study patients.

Clinical Characteristics	NW		OB	
	Females n = 26	Males n = 29	Females n = 25	Males n = 28
Pre-pregnancy BMI (kg/m^2^)	22.77 (18.3-24.9)	22.43 (19.2-24.7)	36.49* (30.03-43.8)	35.78* (29.0-45.5)
Maternal Age (years)	31.60 (24-41)	32.70 (23-46)	31.32 (22-43)	30.40 (20-41)
Gestational age (weeks)	39.32 (39.0-39.7)	39.23 (39.0-39.6)	39.305 (39.0-39.8)	39.24 (39.0-40.0)
Fetal Birth Weight (g)	3477 (2370-4975)	3448 (2845-3941)	3346 (2865-3980)	3392 (2220-4305)
Placenta Weight (g)	581.94 (457.4-758.6)	512.39 (273.6-649.9)	530.53 (389-719.7)	549.13 (349-762.6)
Maternal Weight Gain (kg)	16.65 (6.1-30.7)	13.02 (-3.2-20.4)	11.25* (0.7-45.4)	10.23 (-5.5-20.9)

Maternal and cord plasma samples were collected from male and female offspring of normal-weight (NW) and obese mothers (OB). BMI, body mass index. Data are presented as median (range). *, p<0.05 OB vs. NW group with same fetal sex.

The bold values are statistically significant.

### Effect of Maternal BMI on Fetal and Maternal Circulating Vitamin D

**Figure 1:**
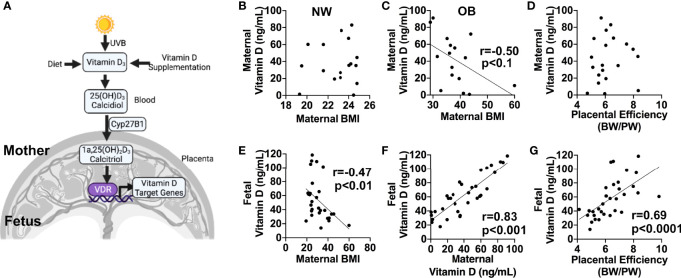
**(A)** Simplified diagram showing pathways for Vitamin D synthesis, bioactivation, and function. Created in Biorender.com. **(B–G)**, 1α,25-dihydroxyvitamin D_3_ (calcidiol) levels in maternal and cord blood plasma from normal-weight (NW) and obese (OB) women, measured by ELISA. **(B–D)**, Correlations between maternal blood plasma Vitamin D concentration and maternal pre-pregnancy BMI in normal-weight **(B)** and obese **(C)** women, and placental efficiency calculated as the ratio between birth weight (BW) and placental weight (PW) **(D)**. **(E–G)**, Correlations between cord blood plasma vitamin D levels and maternal pre-pregnancy BMI **(E)**, maternal plasma Vitamin D **(F)**, and placental efficiency **(G)**. Sample sizes are given in **Table 1**.

Inactive Vitamin D (VitD) is synthesized primarily in human skin and converted to its active form of 1α,25-dihydroxyvitamin D_3_, or calcitriol, in the kidney, placenta, and other target organs by 25-hydroxyvitamin D-1 alpha hydroxylase (CYP27B1) (31) (**Figure 1A**). The actions of calcitriol are mediated by the Vitamin D receptor (VDR), a ligand-dependent nuclear receptor. As circulating levels of VitD are reported to be decreased in obese individuals (32), we hypothesized that Vitamin D status could also be affected by maternal obesity. In the group of normal-weight (NW) women, we observed no correlation between maternal plasma VitD levels and maternal BMI (**Figure 1B**). However, an inverse correlation between maternal circulating VitD and maternal BMI was present in OB women (r=-0.5, *p*<0.1), independent of fetal sex (**Figure 1C**). In addition, male and female cord blood levels of VitD inversely correlated with maternal BMI (r=-0.47, *p*<0.001), but showed positive correlation with maternal plasma VitD (r=0.83, *p*<0.000; **Figures 1E, F** **)**. We also observed a significant correlation between cord blood levels of VitD and placental efficiency, calculated as the ratio of fetal and placental weights (r=0.69, *p*<0.0001, **Figure 1G**). No relationship, however, was observed between maternal blood VitD concentrations and placental efficiency (*p*=0.35, **Figure 1D**).

### Placental Expression of CYP27B1 Is Not Affected by Maternal Obesity

CYP27B1 is the sole Vitamin D 1α-hydroxylase responsible for the last activation step to produce the fully-active VitD hormone calcitriol (31). Since VitD levels were decreased in maternal and cord blood of obese women, we wanted to understand if the placenta plays a role in this process. We began by measuring protein levels of CYP27B1 in placentae from NW and OB women. As shown in **Supplemental Figures 1A, B**, we observed no differences in CYP27B1 expression between the groups. However, within the OB group, levels of CYP27B1 were significantly lower in placentae of female offspring vs. males. Since the placenta is composed of multiple cell types, we next measured expression of CYP27B1 in primary trophoblasts isolated from NW and OB women. We found no statistically significant differences between groups or between sexes (**Supplemental Figures 1C, D**).

### Vitamin D Receptor Protein Expression Is Decreased in Placentae From Obese Women

**Figure 2:**
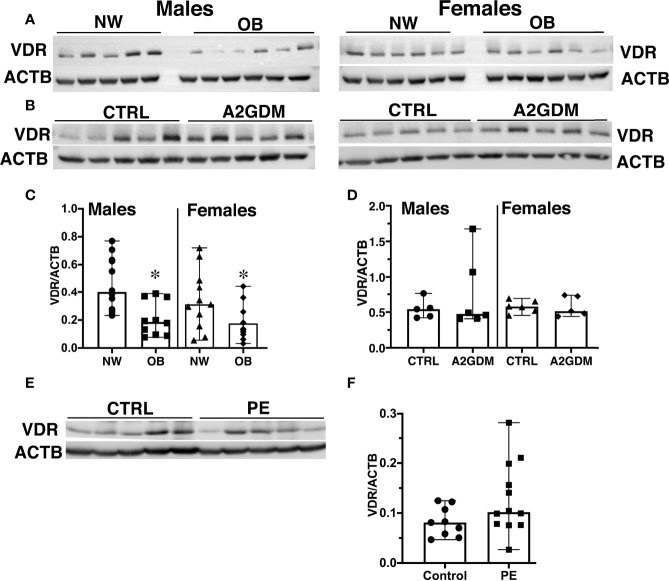
Effect of maternal obesity, gestational diabetes, and preeclampsia on placental expression of Vitamin D receptor (VDR). **(A, C),** Representative images **(A)** and quantification **(C)** by Western blot of VDR in placentae from normal-weight and obese women with male and female fetuses. **(B, D)**, Representative images **(B)** and quantification **(D)** by Western blot of VDR in placentae from pregnancies complicated by gestational diabetes regulated by insulin (A2GDM). **(E, F)**, Representative images **(E)** and quantification **(F)** by Western blot of VDR in placentae from pregnancies complicated by preeclampsia (PE). Control samples for A2GDM and PE were matched by BMI. Data were normalized to β-actin; values are mean ± SEM.*, *p*<0.05. Sample sizes are given in **Table 1** and **Supplemental Tables 1, 2**.

The genomic functions of VitD are mediated through its binding to the Vitamin D receptor (VDR) in target tissues (33). It has been estimated that binding of calcitriol to VDR regulates the expression of about 2000 genes involved in numerous intracellular functions (34). We next hypothesized that the reduced circulating VitD in OB women is a result of decreased VDR expression in the placenta. Western blot analysis of placental tissue from NW and OB women showed a significant 50% reduction in VDR expression in OB vs. NW women, whether having male (*p*<0.001) or female (*p*<0.1) fetuses (**Figures 2A, C**).

Vitamin D deficiency in pregnant women is associated with increased risk for pregnancy complications (35) such as gestational diabetes mellitus (GDM) (36) and preeclampsia (37). We next examined VDR expression in placentae from pregnancies complicated by gestational diabetes regulated by insulin (A2GDM) and preeclampsia (PE). To rule out an effect of maternal adiposity, control samples were matched by maternal BMI. Clinical characteristics of the A2GDM and PE groups and corresponding controls are presented in **Supplemental Tables 1 and 2 (S1 and S2)**. The A2GDM women were slightly older compared to the control group, whether with male or female fetuses (*p*<0.01, **Supplemental Table S1**). Since the C-sections in pregnancies complicated by preeclampsia were performed prior to term, there were significant differences in gestational age and fetal birth weight in the PE group relative to normotensive controls (**Supplemental Table S2**). Nonetheless, when stratified by maternal pre-pregnancy BMI, no statistically significant differences in VDR expression were seen in placentae from women with A2GDM or PE vs. corresponding control groups (**Figures 2B, D–F**).

### Treatment With Vitamin D Increases VDR levels in Primary Trophoblast Cells

**Figure 3:**
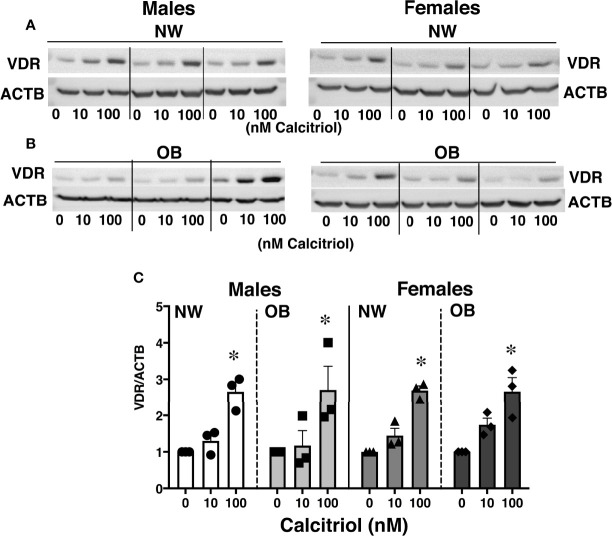
Effect of 24-hour treatment with calcitriol, an active form of Vitamin D, on expression of VDR in primary trophoblasts isolated from placentae of NW and OB women. Representative Western blots **(A, B)** and quantification data **(C)** demonstrating a dose-dependent response to treatment. Data were normalized to β-actin (ACTB) and expressed as fold-change relative to untreated cells. *, *p* < 0.05. N=3-5/dose/group/fetal sex.

Given ongoing debate concerning the efficacy of VitD supplementation and the proper dosage for optimal health during pregnancy (38), we conducted *in vitro* studies to determine the effect of VitD supplementation on placental function. We treated cytotrophoblasts (CTBs) with calcitriol, an active form of VitD, in concentrations previously reported to be effective in cell culture studies using various types of cells, including trophoblasts (39–41). Measuring VDR expression in calcitriol-treated trophoblasts revealed a dose-dependent increase over the 24 hours following treatment, independent of fetal sex and maternal adiposity (**Figure 3**).

### Vitamin D Improves Mitochondrial Respiration in Primary Trophoblasts

**Figure 4:**
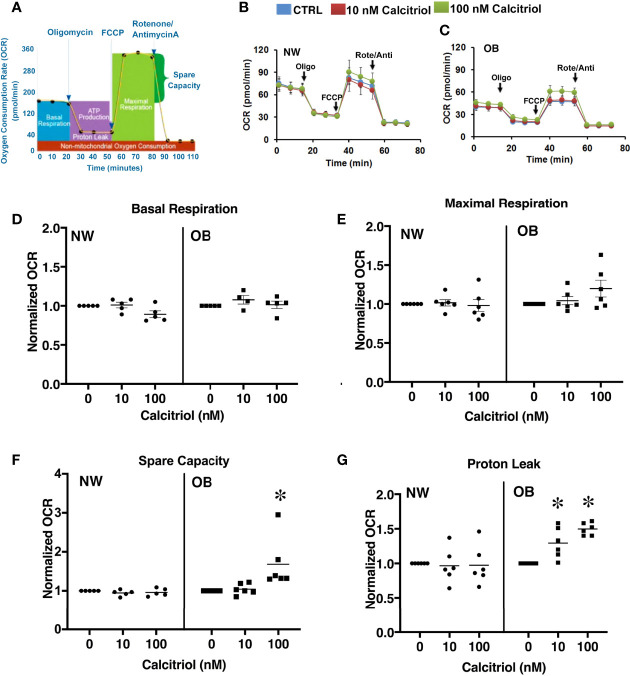
Dose-dependent effect of 24-hour treatment with calcitriol on mitochondrial function in cytotrophoblasts isolated from placentae of NW and OB women. Data from males and females were combined. **(A)** Flowchart of the Seahorse XF Cell Mito Stress test; **(B, C)** Representative curves. **(D)** Basal respiration. **(E)** Maximal respiration. **(F)** Spare capacity. **(G)** Proton leak. *, *p *< 0.05, N=6-7.

It has been previously reported that VitD supplementation of deficient individuals improves mitochondrial function (42). Reports from our group indicate mitochondrial dysfunction to occur in trophoblasts isolated from the placentae of obese women (16). We therefore next aimed to determine whether VitD supplementation would be sufficient to improve mitochondrial respiration in primary trophoblasts isolated from NW and OB women. To this end, trophoblasts were either left untreated or treated with 10 or 100 nM of calcitriol. Oxygen consumption rates were measured using a Seahorse XF Analyzer (**Figure 4**). No changes associated with calcitriol treatment were observed in trophoblasts isolated from placentae of NW women. However, treated trophoblasts from OB women showed significant improvements in spare capacity (**Figure 4F**) and proton leak (**Figure 4G**), independent of fetal sex.

### Vitamin D Reduces Expression of the NLRP3 Inflammasome in CTBs From Obese Women

**Figure 5:**
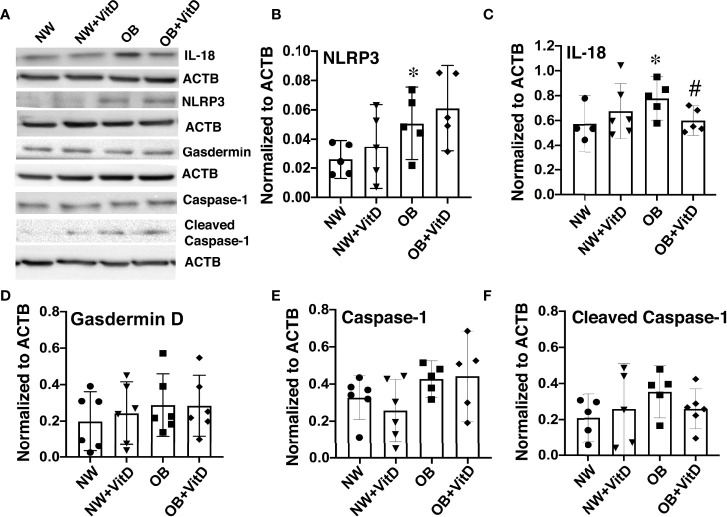
Effect of 24-hour calcitriol treatment on protein expression of NLRP3 inflammasome and the downstream inflammasome activation and pyroptosis factors IL-18, gasdermin D, and caspase-1 in trophoblasts isolated from placentae of normal-weight and obese women. Data from males and females were combined. **(A)** Representative Western blots. **(B)** NLRP3. **(C)** IL-18. **(D)** Gasdermin **(D, E)** Total caspase-1. **(F)** Cleaved p20 caspase-1. Data were normalized to β-actin (ACTB) and expressed as fold-change relative to untreated cells. *, *p* < 0.05. N=6-7. #, p<0.05 vitamin D treated vs. untreated cells.

Vitamin D has been shown to inhibit the NLRP3 inflammasome either by direct binding between NLRP3 and VDR (26) or *via* VDR signaling-mediated inhibition of cytokine secretion (27). We measured the protein expression of NLRP3 and its downstream factors such as gasdermin D, caspase-1, and IL-18 in primary trophoblasts isolated from male and female placentae from normal-weight and obese women, and further assessed the effect of calcitriol in that context. Protein levels of NLRP3 and IL-18 were significantly increased in trophoblasts from OB women vs. NW women (**Figures 5A–C**), whether with male or female fetuses. Calcitriol treatment reduced expression of IL-18 but not NLRP3 (*p*<0.05) in trophoblasts of OB women, independent of fetal sex. No change in IL-18 or NLRP3 expression was detected in trophoblasts from NW women. Likewise, levels of gasdermin D and total and cleaved (p20) caspase-1 remained unchanged across the groups (**Figures 5D–F**).

We also measured expression of NLRP3 and IL-18 in whole placentae collected from NW and OB women, but found no changes associated with maternal adiposity, suggesting that the increases in these proteins were trophoblast-specific (**Supplemental Figure 2**). Importantly, we found that expression of NLRP3 was two-fold higher in female NW placentae and that of IL-18 was two-fold higher in female OB placentae when compared with males within the same maternal BMI group (*p*<0.05).

## Discussion

In this study, we investigated the effect of maternal obesity on maternal and fetal circulating Vitamin D levels, along with the effect of VitD supplementation on VDR expression and on mitochondrial respiration and inflammation in human trophoblasts. Previous studies have shown that people with obesity have less circulating VitD than normal-weight individuals (43). Vitamin D deficiency during pregnancy has been linked to adverse health outcomes including preeclampsia (44), gestational diabetes mellitus (36), increased risk for a cesarean section (45), and preterm birth (46). Maternal VitD deficiency has also been linked to fetal growth restriction (47), congenital heart defects (48), type 1 diabetes mellitus (49), schizophrenia (50), and weak bones in offspring (51). The offspring of mothers who had low circulating VitD concentrations during late pregnancy have been reported to have reduced bone mass at nine years of age (51). Vitamin D has been also shown to play an important role in regulating immune responses during gestation (52).

### Vitamin D in Maternal and Cord Plasma

The only source of Vitamin D for a fetus is the mother, who transfers it across the placenta (53). Maternal 25-hydroxyvitamin concentrations are higher than fetal concentrations, and this metabolite crosses the placenta in relatively large quantities. In agreement with previous studies (54), we found a strong positive correlation between maternal and cord plasma VitD concentrations in both male and female offspring. Bodnar et al. (44) have previously reported a two-fold increase in maternal and neonatal VitD deficiency as maternal BMI increases from 22 to 34 kg/m^2^; we also observed a reduction in maternal plasma VitD levels with increased maternal adiposity, but only in the group of women with BMI higher than 30. The reason for high within-group variability in VitD levels could be that plasma VitD concentrations show seasonal variation, being significantly higher in samples collected during warmer months (April-September) than in winter (55). Due to the smaller sample size in this study, we were unable to factor seasonal periodicity into our data analysis. Another reason could be confounding from VitD supplementation, as maternal plasma concentrations are known to be significantly higher in mothers who receive VitD with their prenatal vitamins.

Our data indicate that in both male and female offspring, there is a significant decrease in cord blood levels of calcidiol with increased maternal BMI. While we observed no relationship between maternal or fetal VitD levels and birth weights (not shown), we found that cord blood VitD deficiency in obese women was correlated with decreased ratio between fetal and placental weights, suggesting placental insufficiency. These findings are in agreement with previously published data showing placental insufficiency in two different mouse models of gestational VitD deficiency (56).

### VDR Expression in the Placenta

The physiologically-active Vitamin D metabolite, 1a,25(OH)_2_D_3_ or calcitriol, does not readily cross the placenta; this is addressed through placental expression of the enzyme CYP27B1, which hydroxylates the inactive form that does cross, 25(OH)D_3_ or calcidiol, to the active form (54). In a study undertaken in a small cohort of 70 pregnant adolescents (<18 years of age) with normal BMI and their term neonates, O’Brien et al. showed a significant correlation between maternal calcidiol and placental expression of CYP27B1, suggesting a link between substrate availability and placental production of calcitriol (57). We, in contrast, found no difference in placental CYP27B1 expression between normal-weight and obese women. This could be explained by differences in clinical characteristics of participants including maternal age (adolescent vs. adult), and maternal pre-pregnancy BMI (all lean vs. lean and obese).

Calcitriol is the major active ligand of the Vitamin D receptor (VDR), a nuclear steroid hormone receptor that acts as a transcriptional activator (58) but can also exert rapid non-genomic effects that influence processes such as cell proliferation and differentiation and apoptosis; these are probably realized *via* VDRs located within the plasma membrane (59). Placentae from obese mothers have lower levels of VDR than those from normal-weight mothers, which means that less active VitD can bind to and be utilized by the organ. Vitamin D deficiency in the placenta has previously been associated with adverse health outcomes including preeclampsia, gestational diabetes, increased risk of having a C-section delivery, and bacterial vaginosis (59). However, in our study, no differences in VDR expression were observed in maternal BMI-matched placentae, whether from normal pregnancies or complicated by preeclampsia or gestational diabetes. This potentially suggests that maternal adiposity is a key determinant in dysregulation of placental VitD metabolism.

### Treatment of CTBs With Vitamin D

Our data show that treatment of cytotrophoblasts with calcitriol increases VDR expression independent of maternal adiposity and fetal sex. We observed a dose-dependent response in trophoblast VDR concentration after short-term treatment with calcitriol, indicating a relationship between VitD levels and *de novo* translation or reduced degradation of VDR; the mechanism underlying this relationship is not yet understood. While the increased presence of VDR in CTBs upon treatment with the active form of VitD may conceptually seem beneficial to overall placental health, it has also been shown that high concentrations of VitD can induce apoptosis (60). Therefore, more research must be done regarding what concentration of prenatal VitD supplementation is optimal to reduce deficiency and increase VDR production while avoiding harm to maternal and fetal cells.

### Mitochondrial Respiration in CTBs Treated With Vitamin D

We have previously reported reduced placental mitochondrial respiration in pregnancies complicated by maternal obesity (16). Since a growing body of evidence suggests that VitD supplementation in deficient individuals improves measures of mitochondrial function (61, 62), we investigated the effects of calcitriol on mitochondrial respiration in trophoblasts. No clear responses of trophoblast mitochondrial respiration to VitD supplementation were seen in either female or male offspring of normal-weight mothers. In contrast, calcitriol-treated trophoblasts from placentae of obese women showed increased spare capacity and proton leak.

In fact, recent studies have confirmed that Vitamin D regulates oxidative capacity through binding of calcitriol to VDRs in skeletal muscle (63). Furthermore, mitochondrial ATP production has been shown to be significantly reduced in VDR-deficient C2C12 myoblasts. These results were recapitulated by an *in vivo* experiment in VitD-deficient mice, which exhibited decreased maximum oxidative capacity (63). Ryan et al. (64) explored the effect of VitD supplementation on human skeletal muscle cells and found that calcitriol administration increases the oxygen consumption rate. Meanwhile, a mouse study similarly looking at the effect of VitD supplementation on mitochondrial function found that treatment with calcitriol leads to inhibition of oxygen consumption, maximal respiration, and proton leak in brown adipocytes (65). Given these disparate findings, future research will reveal potential crosstalk between tissue-specific VDR expression, different VitD doses, and mitochondrial respiration. In our study, the isolation and purification of cytotrophoblasts from term placentae was performed using media with 25 mM glucose, consistent with previous reports (66–68). Further studies are needed to determine the effect of culture conditions, and particularly glucose levels, on the trophoblasts’ response to Vitamin D supplementation.

### Vitamin D Supplementation and Trophoblast Inflammation

Vitamin D is thought to play a role in reducing inflammation and infection in the placenta (69), and has previously been shown to reduce lipopolysaccharide-induced inflammation in the placenta by suppressing placental translocation of the NFκB subunit from cytoplasm to nucleus (70). A mouse model of VitD deficiency revealed immune challenge to elicit a large inflammatory response in the placenta, further underscoring the importance of VitD in placental immune function (52). Inflammasomes are large protein complexes that assemble in the cytosol after activation by pathogen-associated and danger-associated molecular patterns (71). Both direct and indirect interactions have been reported between VitD and the NLRP3 inflammasome. Using immunoprecipitation, Huang et al. demonstrated that VDR forms complexes with NLRP3 in nuclear extracts (26). Rao et al. further demonstrated that binding of the VDR to NLRP3 appears to attenuate deubiquitination of the latter, which is a critical step in inflammasome activation (27). Studies have also shown that calcitriol is capable of inhibiting NLRP3 inflammasome activation and its downstream cytokine signaling in mouse models of liver injury and fibrosis (72), ulcerative colitis (73), and diabetic retinopathy (74). In our study, calcitriol-treated trophoblasts showed no change in NLRP3 expression, but exhibited significantly reduced expression of IL-18. Interestingly, a recent study in COVID-19 patients observed a strong inverse correlation between VitD levels and cytokine production, suggesting a potential role of VitD in reducing complications attributed to the cytokine storm and unregulated inflammation (75).

In summary, receiving adequate amounts of Vitamin D is highly important for maintaining normal placental function in the setting of maternal obesity. We observed beneficial effects of VitD supplementation on VDR expression, mitochondrial respiration, and inflammation in trophoblasts from placentae of obese women. It is unclear whether or not maternal VitD deficiency in obese women is directly responsible for placental dysfunction, but work is underway to evaluate this potentially important phenomenon. Several published reports suggest that VitD controls about 3% of the human genome (76–79), and maternal and fetal deficiencies in VitD could potentially lead to genetic and metabolic abnormalities that emerge later in offspring life. If this is the case, future studies will need to address the important question of when in gestation supplementation with Vitamin D is particularly beneficial in improving placental function, and thus offspring health.

## Data Availability Statement

The original contributions presented in the study are included in the article/**Supplementary Material**. Further inquiries can be directed to the corresponding author.

## Ethics Statement

The studies involving human participants were reviewed and approved by Institutional Review Board of Oregon Health & Science University. The patients/participants provided their written informed consent to participate in this study.

## Author Contributions

EP, NH, MB and AM conceived and planned the experiments; EP, NH, and MB, carried out the experiments; MB and EP collected and processed human samples; NH and AM wrote the manuscript. All authors provided critical feedback and helped shape the research, analysis and manuscript. All authors contributed to the article and approved the submitted version.

## Funding

This work was funded by M. J. Murdock Charitable Trust (to NH), and NIH/NICHD grants HD097398, HD076259A, American Heart Association GRNT29960007 (to AM).

## Conflict of Interest

The authors declare that the research was conducted in the absence of any commercial or financial relationships that could be construed as a potential conflict of interest.

## Publisher’s Note

All claims expressed in this article are solely those of the authors and do not necessarily represent those of their affiliated organizations, or those of the publisher, the editors and the reviewers. Any product that may be evaluated in this article, or claim that may be made by its manufacturer, is not guaranteed or endorsed by the publisher.
