# Supplementary material for: Vitamin D Supplementation Improves Mitochondrial Function and Reduces Inflammation in Placentae of Obese Women
Source: Front Endocrinol (Lausanne). 2022 May 31;13:893848. doi: 10.3389/fendo.2022.893848 (PMC9195071; doi:10.3389/fendo.2022.893848)
Supplement: Supplementary file 1 [file DataSheet_1.docx]

Supplementary Material

**Vitamin D Supplementation Improves Mitochondrial Function and Reduces Inflammation in Placentae of Obese Women**

Elysse Phillips^1^ ^†^, Nora Hendricks^1†^, Matthew Bucher^2^, and Alina Maloyan^1#^.

^1^Knight Cardiovascular Institute, Oregon Health & Science University, Portland, OR, USA

^2^Department of OB/GYN, Oregon Health and Science University, Portland, OR, USA

**^#^Corresponding Author:** Alina Maloyan, Knight Cardiovascular Institute, Oregon Health & Science University, 3181 SW Sam Jackson Park Rd, Portland, OR, USA.

Email: [maloyan@ohsu.edu](mailto:maloyan@ohsu.edu); phone: 503-346-1115.

^†^ These authors have contributed equally to this work.

**Supplemental Table 1.** Clinical characteristics of study patients. Placental samples were collected from male and female offspring of normal pregnancies and pregnancies with insulin-regulated A2GDM. BMI, body mass index. *, *p*<0.05; ^#^, *p*<0.1 in A2GDM group vs. control group within the same fetal sex; ^, *p*<0.05 males vs. females within the same study group.

|  | **Control** | | **A2GDM** | |
| --- | --- | --- | --- | --- |
| Clinical Characteristics | Females  n=6 | Males  n=5 | Females  n=6 | Males  n=5 |
| Pre-pregnancy BMI (kg/m^2^) | 31.06  (28.87-37.03) | 31.65  (25.66-38.04) | 31.50  (26.63-37.90) | 32.02  (25.03-39.60) |
| Maternal Age (years) | 28.8  (27-32) | 27.8  (26-30) | **34.7^#^**  (24-43) | **35.4^#^**  (25-42) |
| Fetal Birth Weight (g) | 3621  (2696-3996) | 3690  (2983-4103) | 3434  (3135-3844) | **3904^**  (3456-4229) |
| Maternal Weight Gain (kg) | 14.78  (10.00-19.10) | 7.62^  (1.40-16.30) | 10.08  (6.30-16.30) | **8.38***  (4.50-12.2) |

**Supplemental Table 2.** Placental samples collected from normotensive women and women with preeclampsia (PE). BMI, body mass index. *, *p*<0.05; ^#^, *p*<0.1 in PE group vs. control group.

| **Clinical Characteristics** | **Control**  **N=9** | **PE**  **N=12** |
| --- | --- | --- |
| Males/Females | 4/5 | 6/6 |
| Pre-pregnancy BMI (kg/m^2^) | 29.00  (22.90-36.30) | 30.70  (21.00-38.40) |
| Gestational Age (weeks) | 39.30  (31.40-40.30) | **34.00^**  (38.90-39.70) |
| Maternal Age (years) | 28  (24-32) | 28  (19-36) |
| Fetal Birth Weight (g) | 3289  (2696-3770) | **1970^**  (1300-2130) |
| Maternal Weight Gain (kg) | 7.71  (-3.17 – 19.05) | 8.94  (0.90-10.43) |

**Supplemental Figure 1.** Protein expression of CYP27B1 in placental whole cell homogenates and isolated trophoblasts from NW and OB women. **(A-B**), Representative images (**A**) and quantification data (**B**) from Western blots for CYP27B1 in whole placentae. (**C-D**), Representative images (**C**) and quantification data (**D**) from Western blots for CYP27B1 in primary cytotrophoblasts. Data were normalized to β-actin (ACTB). ^#^, *p*<0.05 females vs. males within the same maternal BMI group. N=6/sex/maternal BMI.


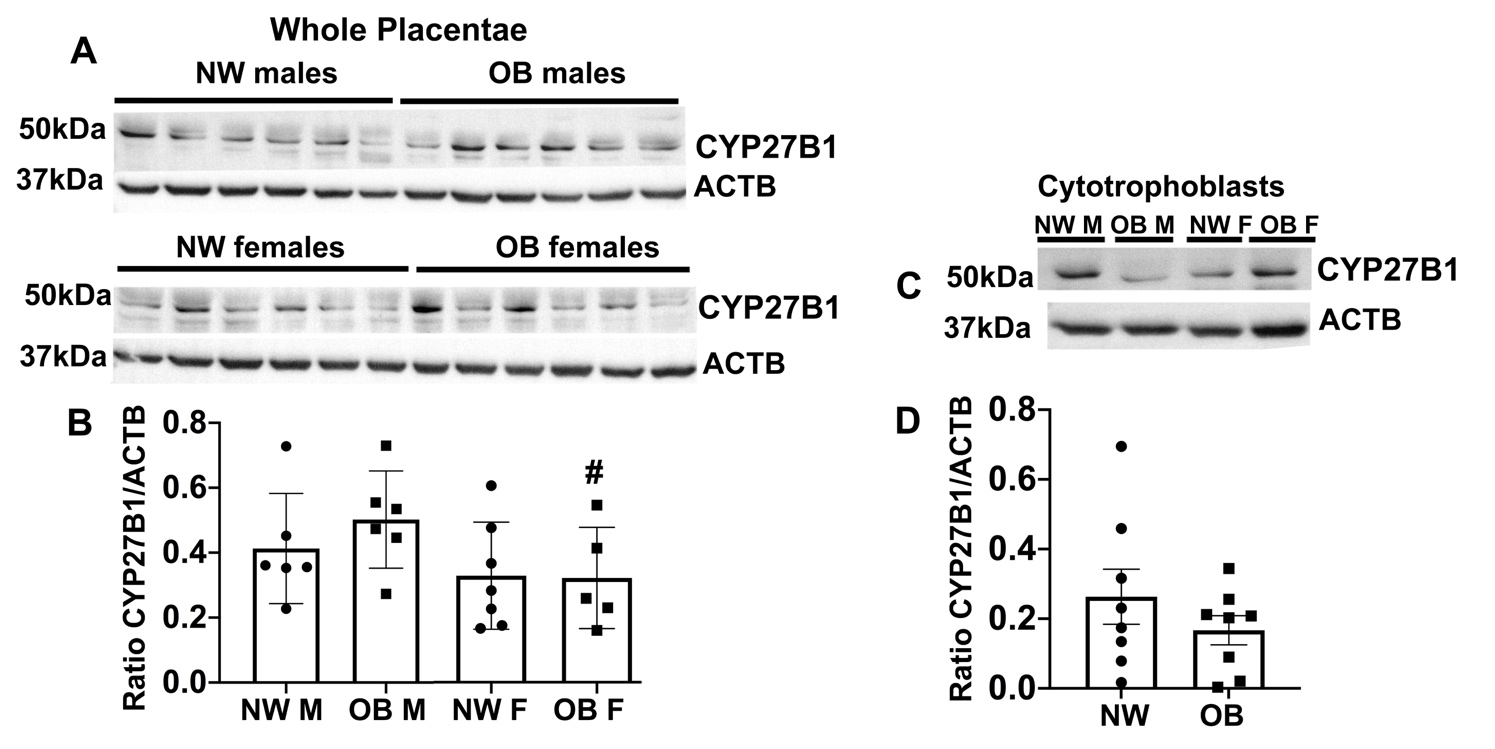


**Supplemental Figure 2.** Protein expression of NLRP3 and IL-18 in whole cell homogenates of placentae from NW and OB women. **(A-C**), Representative images (**A**) and quantification data from Western blots for NLRP3 (**B**) and IL-18 (**C**). Data were normalized to β-actin (ACTB). ^#^, *p*<0.05 females vs. males within the same maternal BMI group. N=5-6/sex/maternal BMI.

**
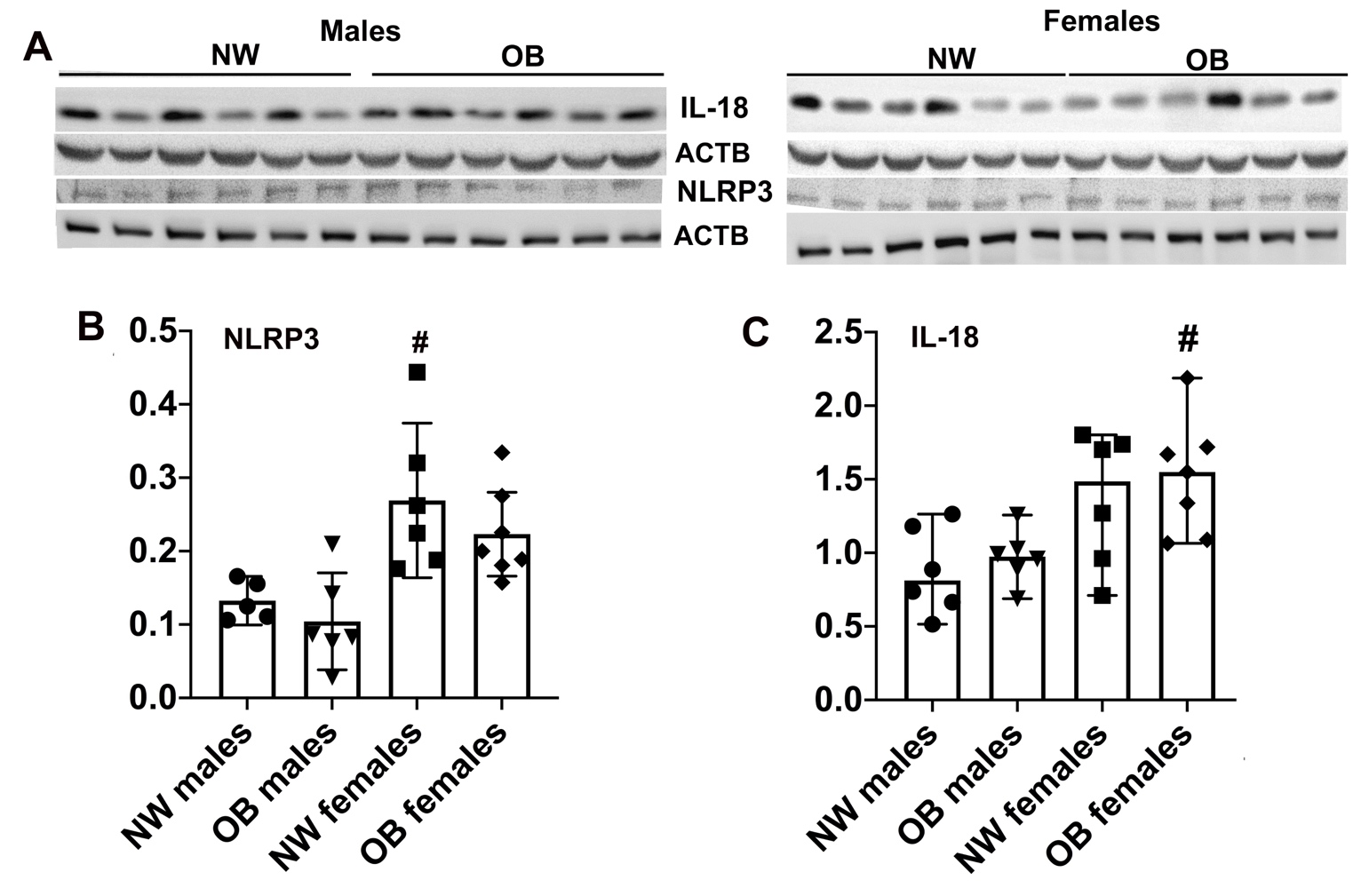
**
